# What Is There to Gain? A Systematic Review on Psychosocial Co-Benefits of Climate Policies

**DOI:** 10.25646/14469

**Published:** 2026-11-04

**Authors:** Ahlke Kip, Mira Tschorn, Susanne Vogel, Michèle D. Birtel, Carla L. Hendricks, Anne Voigt, Silas Rooß, Julia Asbrand

**Affiliations:** 1 University of Freiburg, Medical Psychology and Medical Sociology, Faculty of Medicine, Freiburg, Germany; 2 Freie Universität Berlin, Division of Clinical Psychological Intervention and Division of Health Psychology, Berlin, Germany; 3 German Center for Mental Health (DZPG), partner site Berlin-Potsdam, Germany; 4 MSH Medical School Hamburg, Department of Psychology, Faculty of Human Sciences, Hamburg, Germany; 5 MSH Medical School Hamburg, ICAN Institute for Cognitive and Affective Neuroscience, Hamburg, Germany; 6 London and South East University Group, Institute for Lifecourse Development, London, UK; 7 Social and Preventive Medicine, Department of Sports and Health Sciences, Intra Faculty Unit ‘Cognitive Sciences’, Faculty of Human Science and Faculty of Health Sciences Brandenburg, Research Area Services Research and e-Health, Potsdam University, Brandenburg, Germany; 8 Friedrich-Schiller-University Jena, Department of Psychology, Clinical Psychology of Childhood and Adolescence, Jena, Germany; 9 German Center for Mental Health (DZPG), partner site Halle-Jena-Magdeburg, Germany

**Keywords:** Climate change, Climate policy, Psychosocial health, Mental health, Social cohesion, Well-being

## Abstract

**Background:**

Climate change poses major risks to psychosocial health, yet little is known about potential psychosocial co-benefits of climate policies. This systematic review examines (A) psychosocial co-benefits associated with climate policy measures and (B) moderating factors shaping these effects.

**Methods:**

Following PRISMA guidelines, k = 21 studies were identified and analysed using a PICOS framework and narrative synthesis.

**Results:**

Climate policies, particularly air pollution control, environmental taxation, and nature-based urban interventions, were associated with improvements in subjective well-being, mental health, and social cohesion. Effects were moderated by environmental improvements and physical health gains, and varied by socioeconomic status, age, and contexts such as schools or rural vs. urban locations. Access to and actual use of interventions emerged as relevant but understudied mechanisms. Some studies indicated trade-offs, including unequal distribution of benefits and unintended social effects.

**Conclusions:**

Overall, climate policies can generate psychosocial co-benefits, but evidence remains heterogeneous and methodologically limited. Future research should strengthen causal inference, standardise outcome measures, and examine long-term and distributional effects.

**Key messages**

► Climate policies can generate psychosocial co-benefits, especially improvements in subjective well-being, life satisfaction, and stress-related mental health outcomes.
► Social cohesion, trust, and community engagement may improve when policies create accessible, usable, and shared public spaces.
► Physical health gains, particularly through reduced pollution exposure, better thermal comfort, sleep, and housing conditions, appear to be key pathways to psychosocial co-benefits.
► Effects are not evenly distributed: benefits vary by socioeconomic status, age, gender, vulnerability, and urban or rural context.
► The evidence base remains heterogeneous and methodologically limited, highlighting the need for stronger designs, validated outcomes, and long-term, equity-focused analyses.

## 1. Introduction

Human-induced climate change is one of the greatest challenges of this century. Beyond its ecological impacts, it poses numerous risks to human physical and psychosocial health [1, 2]. Simon Stevens, the former CEO of the UK National Health Service (NHS), described it as the ‘most profound long-term threat to the health of the nation’ [3, para. 5]. Given the severity of these risks, the term climate crisis is adopted here.

The climate crisis contributes directly to humanitarian emergencies – including wildfires, heatwaves, floods, and tropical storms – that are increasing in frequency and intensity [4]. According to the Intergovernmental Panel on Climate Change, between 3.3 and 3.6 billion people already live in areas highly vulnerable to climate change [4]. The World Health Organization (WHO) estimates that climate change will cause an additional 250,000 deaths per year between 2030 and 2050, primarily due to malaria, diarrhoea, malnutrition, and heat stress [5]. Environmental degradation – through rising sea levels, extreme weather events, and changing temperature and precipitation patterns – undermines the social and ecological foundations of health, exacerbates inequalities, and limits access to essential services.

### 1.1 Psychosocial health and the climate crisis

Psychosocial health is a multidimensional construct encompassing mental, social, spiritual, and material well-being, e.g. [6–8]. Building on this literature, psychosocial health can be understood as a dynamic state, shaped by the interaction between individuals and their social environments [8, 9]. In our current research, we adopt a definition of psychosocial health, which describes it as changing over time and existing on a spectrum from healthy to ill [9]. It includes a person’s experiences, perceptions, mental processes, behaviour, and lifestyle. It is closely linked with social relationships and broader social structures. A functioning body and consciousness enable interaction with the social environment. Psychosocial health can be strengthened by resources and support (e.g. emotional support) or weakened by stressors (e.g. work-related stress), with the individual being the central locus of this health. Thus, we apply a positive definition of psychosocial health as (1) mental well-being, including functioning in everyday life (e.g. sleep); (2) the absence of mental distress; and (3) social health, i.e. an individual’s connection with others and participation in all relevant aspects of life.

The impact of the climate crisis on mental health is increasingly recognised. For example, the Intergovernmental Panel on Climate Change acknowledges several associated mental health challenges and negative impacts on well-being [4]. The climate crisis affects psychosocial health through multiple pathways [10]. First, extreme weather events have direct psychological consequences, including increased rates of depression, anxiety, and post-traumatic stress disorder [10–12]. Second, gradual environmental changes, such as rising temperatures and air pollution, are associated with increased aggression, hospitalisations for mental illness, and cognitive impairments [13–15]. Third, indirect effects, including economic instability, food insecurity, and forced migration, contribute to stress and reduced well-being [4, 16]. Finally, climate anxiety reflects the psychological burden arising from anticipated environmental loss and uncertainty and is significantly associated with mental health outcomes such as depression [17]. Younger populations may be particularly affected by climate anxiety, e.g. [18], though studies find varying levels of related impairment [19]. Other theoretical frameworks highlight community-level pathways, including the erosion of social capital and collective structures. In their causal pathways framework, Berry et al. [20] propose that climate change affects mental health through multiple routes: directly through traumatic climate-related events and indirectly through impacts on physical health as well as disruptions to environmental, social, and community conditions. These effects are further shaped by individual and contextual vulnerability, resulting in an uneven distribution of climate-related mental health impacts across individuals and communities. These impacts are unequally distributed. Vulnerable groups – including children, older adults, women, ethnic minorities, and individuals with low socioeconomic status – are disproportionately affected [5, 10, 21, 22]. This highlights the importance of considering moderators of psychosocial outcomes in climate-related research.

While the negative impact of the climate crisis and other ecological crises relating to overstepping the planet’s boundaries have repeatedly been addressed [23, 24], less focus has been on positive ‘side-effects’ of mitigation policies targeting climate and ecological protection.

### 1.2 Mitigation, adaptation, and co-benefits

Climate policies primarily aim to mitigate emissions and support adaptation to climate impacts [4, 25]. However, beyond these primary goals, such policies may generate psychosocial co-benefits. For example, promoting active transport (e.g. cycling or walking) can improve physical health and reduce psychological distress in addition to their effect on emissions [26, 27]. Likewise, urban green spaces can enhance social cohesion and quality of life [28, 29]. Participatory adaptation strategies may also increase perceived agency and reduce feelings of helplessness [30]. Overall, nature-based interventions have gained increasing attention in the past decade and systematic reviews and meta-analyses show beneficial effects for mental health outcomes [31, 32].

Given the multiple pathways through which the climate crisis undermines psychosocial health, climate policies may not only reduce future harm but actively promote well-being. Despite these indications, the evidence base on co-benefits of climate policies remains limited and fragmented. Existing research has largely focused on physical health outcomes or on the perception and use of environmental features, rather than on the psychosocial effects of policy interventions themselves [33]. Recent reviews and scenario-based studies further suggest that mental health is rarely included in climate-related health impact assessments, e.g. [34]. Similarly, reviews of environmental interventions (e.g. afforestation projects) indicate that psychosocial co-benefits are seldom systematically measured, highlighting a critical gap in the literature [35].

### 1.3 The current study

While the negative impacts of the climate crisis on psychosocial health are well-documented, e.g. [1, 13], there is limited systematic evidence on whether and how the implementation of climate policies contributes to psychosocial well-being. Previous reviews were limited to co-benefits of prospective scenarios [34] or climate adaptation and mitigation strategies that targeted wellbeing as primary outcome [35]. Addressing this gap is particularly relevant in the context of rising global mental health challenges, especially among populations at risk such as children and young people [18, 21, 36]. This review provides a systematic overview of existing research on psychosocial co-benefits of climate policy measures. By synthesising evidence across diverse interventions and contexts, it aims to identify key outcomes, underlying mechanisms, and existing research gaps. Specifically, the review addresses the following research questions:

(A) What psychosocial co-benefits are associated with climate policy measures?

(B) How do moderators – such as age, gender, socioeconomic status, and vulnerability – shape these relationships?

## 2. Methods

### 2.1 Search strategy and selection process

We conducted multi-field searches in the databases Web of Science, PubMed, and PsycInfo. To identify relevant studies, search terms indicative of climate protection measures (e.g. environment* AND policy* OR ‘green infrastructure’) were combined with search terms indicative of psychosocial outcomes (e.g. well-being OR stress), and co-benefits (e.g. gain* OR benefit*) in the keyword search (see Supplementary material Table S1). Besides, we repeated the search with the MeSH term ‘Climatic Processes’ instead of keywords for climate protection measures. The search was restricted to human participants and peer-reviewed articles in English and German given the language proficiencies in the research team. The search included all publications up until November 22, 2024. We furthermore cross-checked references of related reviews [33, 35, 37–40] and searched Google Scholar for articles on psychosocial co-benefits of established climate-protection measures, e.g. Reducing Emissions from Deforestation and Forest Degradation (REDD+), Barcelona’s Superblocks, or the Regeneración Urbana, Calidad de Vida y Salud (RUCAS) [41–43]. All 39,349 hits were uploaded into Rayyan [44] for de-duplication and title-abstract screening.

A random sample of 1,350 hits was assessed by five blinded and independent reviewers each. Because Fleiss’s kappa, a chance-corrected measure of agreement among multiple raters, is sensitive to low inclusion rates [45], agreement was also assessed using the overall agreement rate. The overall intra-class correlation coefficient was 0.42 (0.16 – 0.64 between individual raters). However, the overall agreement rate between all reviewers was high at 93.6 % (95.5 – 97.4 between individual raters), meaning that raters made the same inclusion or exclusion decision in most cases. The remaining 30,482 hits were divided between reviewers with all unclear cases being discussed with at least one other reviewer. Full texts were assessed by two reviewers each with unclear cases being discussed among all five reviewers.

The inclusion criteria according to the PICOS framework (Population, Intervention, Comparison, Outcome, Study design); [46] were: P) human respondents (e.g. residents or stakeholders) of all ages or human-related data (e.g. psychiatry admission data); I) mitigation interventions or adaptation interventions that have an additional mitigation effect with a focus on climate protection (requiring a change of environment instead of maintenance of a status quo); C) studies with or without a control condition; O) psychosocial outcomes assessed as co-benefits with a focus on health indicators versus health determinants (e.g. feelings of safety); S) pre-post, controlled studies, and retrospective studies. We excluded P) outcomes related to e.g. companies or households rather than individuals; I) pure adaptation interventions without concurrent mitigation (e.g. installation of air conditioning), interventions focusing on individual behaviour (e.g. physical exercise, change of nutrition), disaster response programs, interventions that are not related to climatic environments (e.g. school environments); C) n.a.; O) human-related measures that are not directly linked to psychosocial outcomes (e.g. finances, physical health, education), non-human-related outcomes (e.g. air pollution scores), outcomes on use, perception, or experience of natural environments; ineligible operationalisation of psychosocial outcome (e.g. not inviting others home as indicator of social isolation; expert opinion without direct involvement such as NGOs); S) simulation or modelling studies, correlative studies (e.g. measuring closeness to green space with mental health; see Table 1).

### 2.2 Data extraction

Data extraction was completed by one reviewer per study with all data being checked by a second reviewer. Any discrepancies were resolved by consensus. Extracted data included information on the study (e.g. quantitative vs. qualitative data, study design), sample (e.g. sampling strategy, age, gender), intervention (e.g. primary aim, year of implementation), outcome (e.g. psychosocial construct, instrument), and results (e.g. type of data analysis, primary and secondary findings such as on moderators).

## 3. Results

### 3.1 Study selection

The database search yielded 31,832 unique records supplemented by 13 additional studies identified through other sources (mainly reference lists of other reviews or studies). After title and abstract screening, 229 full-text articles were assessed for eligibility, resulting in the inclusion of 21 studies. The study selection process is illustrated in Figure 1.

### 3.2 Study characteristics

#### Sociodemographics

Most studies were conducted in Asia (k = 7), followed by North America (k = 6) and Europe (k = 5), while only a small number originated from South America (k =1) or Oceania (k = 2). Study designs included quantitative (k =12), qualitative (k = 3), and mixed-method approaches (k = 6) and comprised both cross-sectional and longitudinal assessments. Only a minority of studies reported the use of a control group (k = 4). Sample sizes ranged from n =17 [48] to n = 61,100 [49]. Most studies did not report a mean age but instead described participants broadly as adults or youths. Samples predominantly consisted of convenience samples such as students and teachers in schools, e.g. [50], or residents of neighbourhoods, e.g. [51]. Gender distributions ranged from balanced samples to predominantly female samples [52, 53], while several studies did not report participants’ gender, e.g. [54].

#### Methodology

Most studies were conducted from 2010 onwards, with one earlier study published in 1995 [55]. Outcomes covered a range of psychosocial constructs, including subjective well-being or related constructs such as social cohesion or quality of life. Most studies applied an observational or quasi-experimental design and used study-specific items to assess outcomes (e.g. ‘Generally, do you think your life is happy?’, rated on a scale from 1 = not happy at all to 5 = very happy [56]). Assessment methods included self-report measures administered via interviews or questionnaires, e.g. [52, 53], other-reports [57], focus group discussions [43, 55, 57], and behavioural observations [58]. Follow-up periods varied widely across studies, with most studies applying post- assessments directly after the intervention or within a year after.

#### Types of interventions and environmental changes

The included studies examined a diverse set of environmental interventions targeting both the built and natural environment. Several studies focused on housing and building retrofits, including energy-efficiency upgrades and comprehensive green renovations of residential buildings, e.g. [52, 59]. Another group of studies investigated urban greening interventions, such as green schoolyard renovations, e.g. [58], the creation of community gardens, e.g. [60], street tree planting, e.g. [57], green roofs, e.g. [60], and the development of urban green infrastructure including greenways and accessible workplace green spaces, e.g. [43, 51]. A smaller number of studies examined nature-based interventions, including ecosystem restoration projects such as mangrove rehabilitation [61]. Finally, some studies analysed policy and governance interventions, including environmental protection taxes [49], air pollution control policies, e.g. [62], and renewable energy programs [63], as well as one urban mobility intervention involving the implementation of superblocks [43].

### 3.3 Risk of bias and study quality

The methodological quality of the included studies varied considerably. High-quality evidence was provided by quasi- experimental designs (e.g. difference-in-differences, regression discontinuity), which strengthened causal inference, e.g. [62, 64]. However, many studies relied on unvalidated outcome assessments, including single-item measures of psychosocial outcomes, e.g. [53, 62], while several employed qualitative approaches, either as stand-alone designs or through focus groups, interviews, or ethnographic methods, e.g. [43, 55, 57]. A smaller number of studies used validated instruments, such as quality-of-life measures including the Short Form Health-36 (SF-36) [54], the EQ-5D-3L and EQ-5D-5L by the EuroQol Research Foundation [43, 53], and the General Health Questionnaire (GHQ-12) [43]. Some studies applied multi-method designs combining quantitative and qualitative data, e.g. [43, 57], which allowed for a more comprehensive assessment of psychosocial outcomes. Only one cluster-randomised controlled trial on household level was identified.

Methodological quality was additionally assessed using the Quality Assessment with Diverse Studies (QuADS) tool, which is specifically suited to reviews including heterogeneous quantitative, qualitative, and mixed-methods evidence. The QuADS comprises 13 criteria, each rated on a scale from 0 to 3, with 0 generally indicating no mention of the criterion and 3 indicating that the criterion was explicitly described, considered, or addressed. Given the aims of the present review, ratings were applied pragmatically with particular emphasis on the quality of methods used to assess psychosocial outcomes, rather than on all aspects of the broader environmental interventions. Assessments were conducted by one reviewer, with ambiguous cases discussed within the research team, and should therefore be interpreted as a structured and supportive overview rather than a strict hierarchical judgement of study quality.

QuADS ratings indicated overall moderate methodological quality across the included studies, with substantial variation reflecting the heterogeneous evidence base (Table 2). Higher ratings were typically observed in studies with stronger quasi-experimental or longitudinal designs, clearer reporting of procedures, and the use of validated outcome measures, e.g. [51, 54, 62, 64]. Moderate ratings were common among studies using qualitative or mixed-methods approaches that were well aligned with their aims but more limited in sampling strategies or analytic transparency, e.g. [43, 57]. Lower ratings were generally associated with insufficient reporting of recruitment, limited justification of analytic methods, or the use of less rigorous and predominantly descriptive designs. Across studies, research aims and study settings were usually well-described, whereas stakeholder involvement and detailed reporting of recruitment or analytic decision-making were less consistently addressed.

### 3.4 Main findings of co-benefits for psychosocial outcomes

#### Well-being and life satisfaction

Thirteen studies examined the effects of climate-protection interventions on well-being. Well-being was operationalised using indicators such as happiness, life satisfaction, morale, perceived quality of life, positive affect, and perceived psychological functioning [43, 51, 52, 54, 56, 57, 59, 60, 62, 64–67].

Overall, most studies reported positive associations between environmental improvements and well-being-related outcomes. For instance, environmental interventions to reduce excessive temperatures were further associated with improvements in quality of life (t = 3.179, p < .01) and increased sleep duration (t = 2.150, p < .05) [52]. Similarly, several urban greening interventions promoted improvements in perceived well-being and morale. Perceived well-being encompasses positive psychological functioning, including emotional stability, life satisfaction, and the ability to cope with daily demands, e.g. [51], whereas morale reflects an individual’s overall psychological state, including feelings of motivation, satisfaction, and being valued within their environment [57].

In workplace settings, the introduction of visible and accessible green infrastructure was perceived as contributing positively to staff well-being, with employees reporting increased happiness and a greater sense of being valued by their employer. In one study, respondents were asked whether working in an environment with more green spaces makes them happier (1 – 5 scale). Both before and after the intervention, 86 % of respondents selected one of the two most positive response categories. However, the proportion selecting the most positive response category increased by 13 % from 54 % to 67 % (p = .05) [57]. Evidence from neighbourhood and urban-scale interventions, altering the built environment, similarly pointed towards improvements in well- being e.g. [57]. Superblock mobility schemes that reduced traffic and increased pedestrian space, were reported to enhance residents’ perceived well-being and feelings of calmness and pleasure through improved aesthetics, reduced noise and pollution, and increased opportunities for social interaction and outdoor activities [43]. Reductions in air pollution might be a critical mechanism as evidence from policy- based environmental interventions showed that while air pollution was related to reduced happiness and life satisfaction [67], new air pollution governance led to improvements in subjective well-being and self-rated health [62, 66].

However, findings were not entirely consistent. Some studies reported no statistically significant changes in mental well-being despite improvements in self-reported quality of life which can be considered a broader, multidimensional construct not limited to mental health (e.g. pre-post difference p = .226 unadjusted; p = .524 adjusted, controlling for sociodemographic and other factors) [51]. One other study reported small declines in overall well-being scores following environmental changes, although these differences were not statistically tested [57]. Further, one study reported that certain policy measures such as pollutant discharge fees could reduce well-being due to inconvenience in daily life, despite environmental benefits [56]. Overall, evidence suggests net positive but context-dependent effects on well-being, e.g. limited to urban areas [64]. For an overview of all studies see Table 2.

#### Psychopathology outcomes

Nine studies examined the effects of environmental interventions on mental health outcomes, including indicators of emotional distress, perceived stress and anxiety, depressive symptoms, sleep, and cognitive functioning [48, 49, 51–54, 57, 59, 64]. Overall, findings were heterogeneous, with some studies reporting improvements, while others found no significant changes or even increases in symptoms.

Some evidence suggests that environmental improvements may contribute to reductions in stress and emotional distress. For instance, environmental interventions to reduce excessive temperatures were associated with decreased emotional distress (t = - 2.085, p < .05) [52]. Similarly, improvements in housing conditions were linked to reductions in stress and anxiety related to energy insecurity and difficulties paying energy bills [59]. One study further reported improvements in sleep duration and memory following environmental interventions, with increases reaching statistical significance [49]. Qualitative evidence from urban environmental interventions further supported these findings, with participants describing a more relaxed atmosphere and reduced feelings of stress following environmental improvements [59].

However, quantitative evidence across studies was not consistent. One longitudinal, quasi-experimental study found no statistically significant pre-post changes in mental health indicators within intervention groups, including measures of psychological well-being and the number of days with poor mental health [53]. Taken together, the evidence suggests that environmental interventions may contribute to reductions in stress-related mental health outcomes, particularly through improvements in thermal comfort, housing conditions, and neighbourhood environments.

#### Social health outcomes

Eleven studies examined the effects of environmental interventions on social and community outcomes, including indicators such as social interactions, bullying or teasing, neighbourhood climate, and perceived community cohesion [43, 48, 50, 51, 55, 57–59, 61, 63, 65]. Observational studies of schoolyard and neighbourhood interventions indicated increases in positive social interactions after environmental changes. For example, the proportion of children observed interacting with others increased from 63.0 % at baseline to 73.3 % following a green schoolyard renovation, with a corresponding rise in positive interactions (27.1 % to 35.2 %) and only very low levels of negative interactions [50]. Similarly, another observational study reported shifts in the quality of interactions, including a decrease in negative interactions (2.4 % to 1.1 %) and an increase in positive interactions (44.3 % to 48.7 %) following a green schoolyard renovation [58]. Retrospective reports from caregivers and school staff also suggested reductions in teasing and bullying after schoolyard renovations, although these changes were not statistically tested and no further changes were observed from baseline to follow-up assessments [50]. Several studies further reported improvements in perceptions of social climate and community relationships. For instance, one study found a significant improvement in perceptions of neighbourhood climate (t = −2.62, p < .05) and a reduction in overall school bullying (d = 0.405, p < .01), alongside improvements in school-community relationships (d = 0.408, p < .01) [58]. The effect sizes are moderate in size according to Cohen’s conventions [68]. In some cases, perceptions of social benefits such as community strengthening, empowerment, and social recognition were reported to outweigh potential social costs of the intervention [61]. Overall, qualitative findings suggested that environmental interventions may facilitate social interaction by creating shared spaces for social activities and reducing barriers to informal encounters among residents [55].

Taken together, the available evidence suggests that environmental interventions may contribute to improvements in social interactions and perceptions of community cohesion, particularly where interventions create shared public spaces or opportunities for community engagement, although quantitative findings remain heterogeneous across studies.

#### Negative side-effects

Overall, systematic evidence on adverse psychosocial effects of environmental interventions remains scarce, as relatively few studies explicitly examined negative outcomes. In a small number of studies, negative trends were observed; however, these were typically not attributable to the intervention itself. For example, one quasi-experimental study reported a small but statistically significant decline in reported social networks over time (4.0 to 3.9; p < .001), although similar changes were observed in the control group and differences between groups were not significant [51]. Additionally, some findings suggest that effects may vary depending on contextual factors such as region and time-lag to implementation, with both positive and negative associations observed [56]. Qualitative evidence further indicates that environmental interventions can give rise to unintended social tensions or unmet expectations [61].

#### Potential moderators

Only few studies investigated the influence of potential moderators. Age [49, 50, 66] and gender [50, 61] repeatedly appeared as sociodemographically relevant constructs. Further, individuals with higher income were more likely to derive well-being benefits from environmental improvements, whereas lower-income groups prioritised material conditions [67]. However, individual attitudes towards and perceptions of the implemented interventions were also relevant, e.g. [63]. Finally, differences between urban and rural populations suggest that different benefits may emerge depending on the context, with urban residents reporting improvements in subjective well-being, while rural populations showed more pronounced physical health gains [66].

## 4. Discussion

This review aimed to (A) identify psychosocial co-benefits associated with climate policy measures and (B) examine moderators shaping these relationships. Surprisingly few studies have targeted this specific subject to date. Overall, the evidence indicates that climate policies – particularly air pollution control, environmental taxation, and nature-based urban interventions – are associated with positive psychosocial outcomes, although effects are heterogeneous and context-dependent. Across the included studies, environmental interventions were most consistently associated with improvements in subjective well-being and mental health, including reductions in stress, anxiety, and sleep disturbances, as well as increases in life satisfaction and emotional stability [49, 62, 64]. These findings extend prior literature linking environmental quality to well-being [69] by demonstrating that policy-driven environmental improvements can actively enhance psychosocial health. Also, several studies highlight social co-benefits, including increased social cohesion, trust, and community engagement, particularly in the context of community-based or nature-based interventions [63]. These findings suggest that climate policies can generate collective psychosocial benefits, not limited to individual-level outcomes.

Consistent with broader health-well-being frameworks [8], physical health improvements may function as a central mediating pathway. Reductions in pollution exposure are associated with lower morbidity and improved self-rated health, which in turn contribute to higher well-being [49, 62]. However, it has to be considered that several studies found no statistically significant changes in mental health measures, e.g. [51], and in some cases, a slight decline in overall well- being [57] or a decrease in social networks, e.g. [51], was reported. These findings can be understood through a resource-based framework: climate policies may strengthen psychosocial health by improving individual resources such as physical health, social resources such as cohesion and community engagement, and material resources such as housing conditions and reduced economic burden [70]. This aligns with planetary health approaches, which emphasise the interdependence of human well-being, social conditions, and healthy environments e.g. [71].

### 4.1 Potential moderators

Socioeconomic status emerged as a key moderator, although findings were mixed: some studies suggest that individuals with higher socioeconomic resources derive greater psychosocial benefits, potentially due to increased capacity to utilise improved environments [67]. Conversely, other findings indicate stronger health gains among socioeconomically disadvantaged groups due to higher baseline exposure, suggesting differential benefit pathways rather than uniform effects. Older adults appear to benefit substantially from environmental policies, particularly through improvements in physical and cognitive health [49]. Evidence for children and adolescents – primarily from green infrastructure studies – indicates benefits for psychological development and well- being, although these are less frequently examined in policy contexts. Finally, the observed difference in co-benefits for urban and rural residents [49] highlights the importance of contextual and environmental baseline conditions. Several studies indicate that effects vary depending on policy design, implementation intensity, and regional conditions, including non-linear relationships between regulation and well-being [56]. This suggests that psychosocial co-benefits are not inherent to policies per se but depend on how policies are implemented and experienced.

A cross-cutting issue relevant to both research questions is the distinction between access to climate-related interventions and their actual use. Evidence from urban green space research indicates that availability alone does not guarantee psychosocial benefits, which depend on engagement, perceived safety, and usability [72, 73]. This implies that some null or weak findings in the included studies may reflect limited use rather than absence of effect, suggesting an important behavioural mechanism that is insufficiently captured in current research.

Additionally, the identified co-benefits may be incompletely captured due to the predominance of short-term study designs. Climate policies can trigger longer-term systemic changes, such as increased urban attractiveness, migration, and social development, which may further influence well- being over time. At the same time, self-selection processes [74, 75] complicate interpretation, as individuals may sort into environments that match their preferences or resources. Additionally, urban greening may contribute to gentrification and rising housing costs, potentially offsetting benefits for vulnerable populations, e.g. [76, 77]. Finally, certain interventions may impose economic or psychosocial burdens, such as increased labour demands or effects on income inequality, e.g. [78]. These findings indicate that psychosocial co-benefits should be considered alongside distributional and contextual trade-offs.

### 4.2 Methodological considerations

#### Original studies

This review provides a first synthesis of the evidence, indicating that the field of psychosocial co-benefits of climate policies remains in its infancy. While the limited number of high-quality studies restricts the conclusions that can currently be drawn, a systematic synthesis can be particularly valuable at this early stage of the field. It allows for an identification of first indications of potential co-benefits and, importantly, a specification of methodological improvements needed to establish a more robust evidence base in the future. This interpretation is consistent with the QuADS assessment, which indicated predominantly moderate methodological quality across the included studies. Moderate ratings often reflected a reasonable alignment between study aims and methods, particularly in qualitative and mixed-methods studies, but also limitations in sampling strategies, analytic transparency, or reporting detail. Across studies, aims and settings were usually well described, whereas recruitment procedures, stakeholder involvement, and analytic decision-making were less consistently addressed. While negative effects of the climate crisis on both general symptoms of psychopathology and established mental disorders have been described quite regularly [13], more thorough attention has yet to be directed towards preventing and decreasing clinical levels of psychopathology through climate policies. This crucial interdisciplinary topic faces several key challenges that need to be addressed in future research to improve the reliability of its conclusions. First, psychosocial constructs were defined very differently between studies and not always as intended by psychological and health sciences [8]. They were further limited to narrow levels of psychosocial health, e.g. evidence for spiritual or existential dimensions (e.g. meaning, place attachment) has so far been less systematically assessed and remains an area for future research. Second, given that the main goal of the interventions was changes in the environment, assessments of co-benefits were sometimes only added post-hoc in a retrospective manner, e.g., [50], or assessed only indirectly via observation [50, 58], resulting in an enormous heterogeneity of methods. For example, our review focused on individual-level psychosocial health, while several studies assessed this outcome at household level [79, 80]. As differences in reporters’ perspectives are the norm rather than the exception [81, 82], a multi-person approach should be preferred over a single-respondent household perspective. Additionally, outcome measures were often insufficient, with most studies relying on single-item indicators rather than validated, psychometrically robust scales [53, 62]. Third, only a few studies applied mediation analyses to investigate the indirect pathways from environmental changes to psychosocial benefits. For example, climate protection measures could induce a sense of security [83–86], reinstate attention, and increase physical health [4, 30, 65, 86]. Thus, all findings should be interpreted very cautiously and rather serve to generate impulses for future research. Finally, assessments of long-term effects, including follow-up, remain limited [49, 62, 64], although such effects may be particularly important, given that climate protection measures are likely to unfold their impact over the longer term rather than the short term [1, 87, 88].

#### Current study

Regarding limitations of our systematic review, this review was not pre-registered, and inclusion criteria were iteratively refined due to the conceptual diversity of climate-related interventions. Further, given the broad and heterogeneous nature of the field, our review focused exclusively on psychosocial health indicators, without considering health determinants or outcomes at broader societal and somatic levels. Thus, it provides only a first glimpse into the field and presents potential future lines of research.

### 4.3 Implications for research and society

The findings suggest that climate policies can generate psychosocial co-benefits across multiple domains, but these are not uniform and depend on contextual and individual factors. Future research should employ longitudinal and experimental designs [89], systematically assess moderators and vulnerable groups, including a broad perspective on psychosocial health and regions which might suffer even more from negative effects of the climate crisis such as sub-Saharan Africa, e.g. [90, 91]. Of note, none of the studies included here has focused on the continent of Africa. Also, next to indicators for psychosocial health, a broader network of health- related determinants should be considered such as gender empowerment [92], food and nutrition security [93], and days in school/at work [94]. Current evidence also highlights limitations in interdisciplinary integration and measurement consistency, which should be addressed to improve evidence quality. This calls for interdisciplinary research combining expertise on both climate interventions and high-quality psychosocial assessments.

For society and policy, the findings suggest that climate policies may generate benefits beyond environmental and physical health outcomes. Measures that reduce pollution, improve housing or thermal conditions, or provide accessible shared spaces may support everyday well-being and social interaction. While psychosocial outcomes are typically not the primary objective of climate policy, some measures may explicitly pursue well-being or social benefits alongside environmental goals. More generally, psychosocial effects should be considered as potential co-benefits of climate action rather than assumed to occur across all policies. Their assessment should consider accessibility, usability, and the distribution of benefits and burdens across population groups. This highlights the need for standardised frameworks to assess psychosocial impacts, similar to existing approaches in urban health research [91, 95].

## Figures and Tables

**Figure 1:**
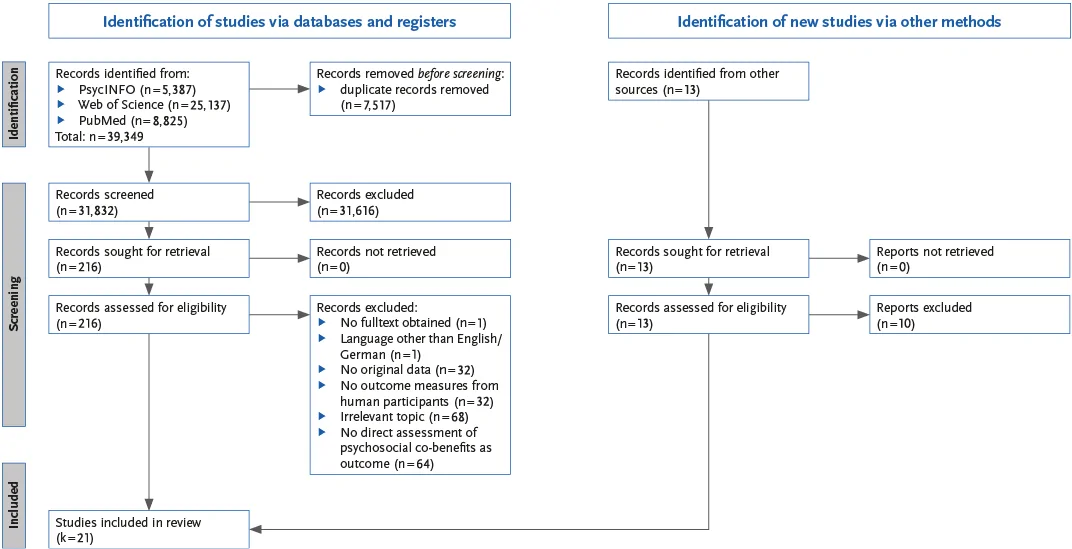
Flowchart of the literature selection process based on PRISMA. Source: Own presentation based on Page et al. [47] PRISMA = Preferred Reporting Items for Systematic reviews and Meta-Analyses

**Table 1:** Overview of all included studies based on PICOS

**Study**	**Population (P)**	**Intervention/ Exposure (I)**	**Comparator (C)**	**Psychosocial Outcomes (O)^1^**	**Study Design (S)**	**Moderators**	**Results**	**Major limitations**
Ahrentzen et al. (2016) [52]	Older adults, lower income, convenience sample (USA), n = 57	Energy retrofit housing	Pre/post	General health/quality of life, emotional distress, sleep	Quantitative, longitudinal, self-report applied as interview	-	Reduction in excessive temperature associated with improved quality of life/health, reduced emotional distress, and increased sleep duration (all p < .05).	Single-case study, assessment via single-item surveys
Bates et al. (2018) [50]	Children, self-selected by schools, reports by caregivers and teachers (USA), observations T1: n = 3,345, T2: n = 3,710; participants T1: n = 64/33, T3: n = 61/40	Green schoolyard renovation	Pre/post, retrospective	Social interactions, teasing/ bullying	Mixed methods, longitudinal, behavioural observation and self-report	Age, gender	Intervention associated with increased social interaction and more positive interactions; bullying perceived as reduced (not significant). Interaction patterns differed by ethnicity and age (p < .01), with no gender effects.	Retrospective assessments of changes in student safety etc.
Bohnert et al. (2022) [58]	Children, self-selected by schools, reports by caregivers and teachers (USA), observations T1: n =1,713, T2: n =1,937, participants: T1: n = 94/66/27; T2: n = 84/45/25	Green schoolyard renovation	Control schools/ pre-post	Social interactions, social cohesion	Mixed methods, longitudinal, behavioural observation and self-report	School context	Intervention associated with fewer interactions overall but higher proportion of positive interactions and reduced negative/neutral interactions; improved neighbourhood climate (p < .05), reduced bullying and stronger school-community ties (p < .01); heterogeneous effects across schools, some outcomes non-significant.	No report if T1 and T2 respondents were identical
Breysse et al. (2015) [53]	Older adults, lower income, convenience sample (USA), n = 40	Green renovation/ building renovation	Pre/post	General mental health	Quantitative, longitudinal, self-report applied as interview	-	Intervention showed no significant within-group changes, but significant improvements relative to control (which declined) for selected outcomes (p < .05); no effects for other measures.	home conditions of control group unknown, enrolling 13 new tenants at T2, assessment via single-item
Cinderby & Bagwell (2017) [57]	Business community, convenience sample (UK), T1: n = 28, T2: n = 32	Green urban infrastructure	Exposure vs. non-exposure	Enjoyment, well-being, happiness	Qualitative, others-report (manager), self-report, interview	-	Green infrastructure linked to higher reported happiness (p = .05), improved morale, social interaction, and environmental awareness; overall well-being ratings slightly decreased (not tested), with stable social support.	Change scores calculated based on different respondent; other massive development projects conducted parallel, some assessments via single-item
Gill and Simeoni (1995) [55]	Community residents, convenience sample (Australia), n = 55	Environmental enhancement project including urban park development	Pre/post exposure	Communal congeniality	Observational, cross-sectional, interview and focus group discussions	-	Participants reported enhanced community interaction, greater use of shared social spaces, and improved intercultural exchange following the park intervention.	Representativeness of data
Hernández & Phillips (2015) [59]	Low-income households and landlords, convenience sample (USA), n = 20	Energy efficiency improvements of low-income housing units	Pre/post intervention	Stress, quality of relationships, comfort, positive emotions	Mixed methods, cross-sectional, interview	-	Intervention reduced energy- related stress and financial strain, increased well-being, and fostered positive feelings and improved tenant-landlord relationships.	Interviews conducted with household heads instead of single members; discrepancies between perspective of building owners and tenants
Hoffman (2020) [48]	Incarcerated individuals and community members, self-selection (USA), n =17	Green space orchard project in correctional setting	No formal control; post-project survey associations	Community connectedness	Mixed methods, quasi-experimental, cross-sectional, self-report	-	Strong correlations between perceived importance of volunteering and community connectedness, and between continued engagement and prosocial impact (r ≈ .91 – .93, p < 0.001); qualitative data suggests therapeutic benefits and enhanced physical, mental, and social well-being.	small sample; self-selection, no distinction between groups (inmates and community members), self- developed survey
Howden-Chapman et al. (2007) [54]	Low-income households with respiratory vulnerability, convenience sample (New Zealand), experimental: n = 960, control: n = 944	Home insulation retrofit	Randomised control households	General health, social functioning, emotional role functioning happiness	Quantitative, longitudinal, cluster-randomised, self- report	Analyses controlled for sex, ethnic origin, age group	Intervention led to significant improvements in social and emotional functioning (p < .0001), reduced odds of poor mental health (OR ≈ 0.56), and lower likelihood of reporting poor/fair health (OR ≈ 0.50).	Potential argument that household income rather than substandard housing is fundamental problem underlying health issues
Hunter et al. (2021) [51]	Urban adult residents, random selection (UK), T1: n =1,037, T2: n = 968	Urban greenway regeneration	Control areas and distance bands	Quality of life, mental well-being, social networks	Quantitative, longitudinal, self-report as interview	Analyses controlled for age, season,education, car ownership and deprivation	Quality of life declined in overall, but less in the intervention area compared to control (p < 0.001). Mental wellbeing did not change; social networks declined slightly, with no difference between areas.	Local control areas were included but were much smaller than the intervention sample. The planned comparison with Northern Ireland SAPAS was not possible because the follow-up survey was not commissioned
Kim et al. (2018) [60]	Urban residents/stakeholders (South Korea), n = 218	Rooftop farm vs. roof garden on university building	Comparison across roof types	Emotions and well-being	Quantitative, cross-sectional, self-report applied as interview	Individual preferences for intervention	Majority perceived green roofs as needed (> 80 %), with a third reporting restorative use; preference for green roofs associated with higher motivation and satisfaction.	no pre-test, no control group
Marshall et al. (2023) [65]	University/community respondents and urban biodiversity setting, random sampling (UK), n = 278	Urban wildflower meadow replacing lawn	Remaining lawn and pre-sowing baseline	Mental well-being	Mixed methods, cross-sectional, self-report applied as interview	-	Significantly higher mental well-being reported for meadows vs. lawns (83.5 % vs. 45 %, p < .001), with qualitative data highlighting strong restorative and emotional benefits.	Primary focus on biology and climate aspects, low survey quality
Pérez et al. (2025) [43]	Urban residents and workers (Barcelona, Spain), focus groups: n ≈ 20, quantitative study: final n not reported	Superblocks urban redesign	Pre/post, affected vs. control areas, mixed methods	General health, well-being, mental health, social support	Mixed-methods natural experiment, longitudinal and cross-sectional, self-report applied as interview, observational measures, discussion	-	Participants reported improved well-being (~ 45 – 55 %), with qualitative evidence indicating enhanced aesthetics, reduced pollution and noise, increased opportunities for physical activity and outdoor use, and stronger social interaction and neighbourhood cohesion.	Assessment in parallel to COVID-19 pandemic
Richerzhagen et al. (2019) [61]	Rural and peri-urban communities, convenience sampling (Colombia), n = 71	Ecosystem-based adaptation projects	Project vs. nearby non-project experience / qualitative comparison	Perceived social benefits	Qualitative, cross-sectional, self-report applied as individual and group interview, focus groups, observation	Gender	Social benefits outweighed costs, with reported improvements in community cohesion, empowerment, and perceived appreciation; gender differences observed, with stronger empowerment effects among women.	interviewees might be biased by institutional affiliations and social standing, no confidentiality during some interviews, no pre-test, no control group
Song, Li et al. (2019) [66]	Urban residents in Chinese air-pollution transmission channel cities, random sampling (China), n =1,016	Specific air pollution control measures	Policy exposure variation	Subjective well-being, happiness, life satisfaction	Quantitative, cross-sectional, self-report	Income, education, age	Policies targeting air pollution (e.g. heating regulation, EV subsidies) linked to increased well-being; results consistent in robustness analyses.	assessment of well-being and life-satisfaction with only 1 item each
Song, Guo et al. (2019) [56]	General population (China), sample size not reported	Environmental regulations (command-and- control, market- based, informal)	Cross-region comparison	Subjective well-being	Quantitative, cross-sectional, self-report	National region, time-lag	Command-and-control and market-based regulations showed region-specific, non-linear or null associations with well-being; effects varied depending on temporal lag to intervention.	No sample size reported, assessment of subjective wellbeing with only 1 item
Tsagkari et al. (2022) [63]	Island communities, random sampling (Greece and Spain), n = 50/145	Local renewable energy projects including wind, photovoltaic, hydro-wind power	Cross-case/ post-implementation perceptions	Social cohesion	Quantitative, cross-sectional, self-report	Perception on success of the project	Participants reported overall agreement with social benefits; institutional benefits were perceived as stronger, while economic benefits were rated lower than social benefits.	No pre-test, no control group
Wang & Tang (2023) [67]	Representative data, conducted annually (China), sample size not reported	Environmental protection tax and subjective air pollution context	Cross-regional variation	Subjective well-being	Quantitative, cross-sectional, self-report	Income, region, urban- rural, gender, age	Subjective air pollution significantly decreased well-being, while environmental protection tax increased happiness; negative effects were larger for low-income individuals and present in both urban and rural populations.	Other relevant health factors such as work status or education might be responsible for effects
Wang et al. (2024) [49]	Middle-aged and older adults (China), n ≈ 61,100	Environmental protection tax policy	Treatment vs. non-treatment cities over time	Sleep quality, memory condition	Quantitative, longitudinal, self-report	Age, marital status	Significant increase in sleep duration (p < .05) and marginal improvement in memory function (p < .10) following the intervention.	Assessments using only 1 item per construct, environmental protection tax policy involves multiple factors (tax rate settings, collection methods, and the implementation strength of local governments)
Xie et al. (2024) [62]	General population/households, representative survey (China), n = not reported	Large-scale air pollution control policy	Policy boundary and non-policy regions	Happiness, self-rated health	Quasi-experimental, quantitative, longitudinal, observational	Regional location	Air quality improvements linked to increased happiness (β ≈ −0.017, p < .01) and improved perceived health (β ≈ 0.007, p < .01).	reliance on observational data; difficulty measuring morbidity impacts
Zhao & Li (2024) [64]	Working-age adults, random sampling (China), observations in different subsamples n = 9,332-30,918	Atmosphere Ten Articles Plan (ATAP) to target pollution	Staggered city implementation/pre-post	Subjective well-being	Quantitative, longitudinal, self-report applied as interview	Emotional stability, mental health	ATAP significantly increased happiness and likelihood of being very happy (p < .001), with effects mediated by improved mental health, emotional stability, and reduced insomnia; benefits were greater for low-income, urban, and middle-aged/older individuals, with no effects for rural or younger groups.	assessment of subjective well-being with only 1 item

^1^limited to relevant psychosocial co-benefits

PICOS = Population, Intervention/Exposure, Comparator, Outcome, Study design. UK = United Kingdom, OR = odds ratio

**Table 2:** Quality appraisal of included studies based on QuADS criteria

**Criterion**	**1.**	**2.**	**3.**	**4.**	**5.**	**6.**	**7.**	**8.**	**9.**	**10.**	**11.**	**12.**	**13.**
	**Theoretical or conceptual underpinning to the research**	**Statement of research aim/s**	**Clear description of research setting and target population**	**Appropriate study design to address the stated research aim/s**	**Appropriate sampling to address the research aim/s**	**Rationale for choice of data collection tool/s**	**Format & content of data collection tool is appropriate to address the stated research aim/s**	**Description of data collection procedure**	**Recruitment data provided**	**Justification for analytic method selected**	**The method of analysis was appropriate to answer the research aim/s**	**Evidence that the research stakeholders have been considered in research design or conduct**	**Strengths and limitations critically discussed**
Ahrentzen et al. (2016) [52]	2	3	3	2	2	2	3	3	2	2	3	1	3
Bates et al. (2018) [50]	2	3	3	2	2	2	2	2	2	2	3	1	2
Bohnert et al. (2022) [58]	2	3	3	3	2	2	3	3	2	2	3	1	2
Breysse et al. (2015) [53]	1	3	3	1	2	1	3	3	2	1	2	0	3
Cinderby & Bagwell (2017) [57]	3	3	3	3	2	2	3	3	3	2	3	2	3
Gill and Simeoni (1995) [55]	3	3	3	3	2	2	3	3	1	1	2	2	1
Hernández & Phillips (2015) [59]	1	3	3	2	2	2	2	3	2	2	3	2	3
Hoffman (2020) [48]	1	3	2	1	1	1	1	2	1	1	1	1	0
Howden- Chapman et al. (2007) [54]	2	3	3	3	3	2	3	3	3	3	3	1	3
Hunter et al. (2021) [51]	3	3	3	3	3	3	3	3	3	2	3	2	3
Kim et al. (2018) [60]	3	3	3	2	1	0	2	1	0	0	3	0	1
Marshall et al. (2023) [65]	2	3	2	3	1	1	1	1	1	0	3	0	0
Pérez et al. (2025) [43] quantitative	3	3	3	2	1	3	2	2	1	2	3	2	1
Pérez et al. (2025) [43] qualitative	3	3	3	3	2	2	3	3	2	1	3	2	2
Richerzhagen et al. (2019) [61]	3	3	3	2	1	2	1	2	1	0	1	1	1
Song, Li et al. (2019) [66]	3	3	3	3	3	2	1	1	1	3	3	0	1
Song, Guo et al. (2019) [56]	3	2	2	3	2	3	1	3	3	3	3	3	1
Tsagkari et al. (2022) [63]	3	3	3	2	3	3	2	2	1	0	2	1	2
Wang & Tang (2023) [67]	2	3	2	2	1	1	2	1	1	2	2	0	2
Wang et al. (2024) [49]	3	3	3	3	2	1	1	1	1	2	3	0	2
Xie et al. (2024) [62]	3	3	3	3	3	2	3	3	2	3	3	0	3
Zhao & Li (2024) [64]	3	3	3	3	2	3	2	2	1	3	3	1	3

Pérez et al., 2025: rated separately due to distinct subprojects with different methodologies: quantitative components (survey) and qualitative components

(focus groups, ethnographic studies)

QuADS = Quality assessment with diverse studies; the QuADS includes 13 criteria, each rated from 0 to 3. A score of 0 generally indicates that the criterion was not mentioned, whereas a score of 3 indicates that the criterion was explicitly described, considered, or addressed. Higher total scores indicate more complete reporting and/or stronger methodological appropriateness. Cell shading corresponds to the respective QuADS rating and is provided to facilitate visual interpretation across studies and criteria.
